# Ultrasonography as an instrument to evaluate lymphedema secondary to breast cancer: systematic review

**DOI:** 10.1590/1677-5449.202201441

**Published:** 2023-12-18

**Authors:** Laura Ferreira de Rezende, João Paulo Martins Piloni, Vitória Livorato Kempa, Júlia Franco Ramos Silva, Vanessa Fonseca Vilas Boas, Regiane Luz Carvalho, Ângela Gonçalves Marx

**Affiliations:** 1 Centro Universitário das Faculdades Associadas de Ensino - FAE, São João da Boa Vista, SP, Brasil.; 2 Clínica Ângela Marx, São Paulo, SP, Brasil.

**Keywords:** ultrasonography, breast cancer lymphedema, breast neoplasms

## Abstract

Lymphedema is a chronic and progressive disease characterized by fluid accumulation, causing tissue edema as a result of a compromised lymphatic system. Diagnostic ultrasound (DUS) is a method capable of assessing soft tissue characteristics that can be used reliably to diagnose lymphedema as well as for measuring tissue compliance in a clinical setting. This is a systematic review, aiming to evaluate articles that made use of DUS in management of lymphedema secondary to breast cancer. A total of 570 articles were selected, exported to the Rayyan QCRI review program, and then screened by two researchers. From this search, 25 articles were selected after the authors reached consensus and were catalogued as to their main results. Diagnostic ultrasound was identified as an advantageous method that is safe, minimally invasive, low cost, and radiation free and is useful for evaluating the efficacy of therapies used in lymphedema treatment.

## INTRODUCTION

Lymphedema is a severe chronic and progressive disease characterized by a high concentration of fluids containing proteins in the interstitial space, caused by partial or total obstruction of lymphatic drainage, provoking tissue edema, and caused by an impaired lymphatic system. As lymphedema worsens, there is greater involvement of the subfascial lymphatic system than the epifascial lymphatic system. As lymphedema progresses, fibrocytes and/or adipocytes begin to proliferate in affected areas, causing structural changes in skin and subcutaneous tissues and increasing vulnerability to bacterial and fungal infections.^1-3^

One of the most important manifestations of lymphedema is lymphedema secondary to breast cancer (LSBC). Studies report that during the initial stages lymphedema presents clinically as upper limb edema in the area of the arm, shoulder, neck, or trunk ipsilateral to treatment, because of removal of local lymph nodes and lymph vessels, compromising the local lymphatic system and impairing lymphatic drainage. There may be increased protein content in the affected tissues, resulting in chronic inflammation, fibrosis, pain, limited amplitude of movement, and/or paresthesia, in addition to reduced immune function, increasing the risk of local inflammation and infections.^4-7^

It has been demonstrated that lymphedema progresses in the form of a vicious cycle, in which lymphatic stasis provokes development of chronic inflammation, involving uncontrolled macrophage and CD4 + cell response and accumulation of fat, which also promotes chronic inflammation through macrophage infiltration and activation, producing inflammatory cytokines which in turn provoke more lymphatic stasis, reducing lymphatic pumping and increasing capillary filtration.^8^

With the objective of improving patients’ quality of life, reducing their physical and psychological discomfort, it is essential to conduct a precise diagnosis of the problem to achieve better prognosis and support treatment planning. It has been shown that this diagnosis is not always easy to achieve and it is necessary to differentiate it from other pathologies with similar conditions to lymphedema, such as local edema and fibrosis of subcutaneous tissues. There are many tests that can be used with the objective of achieving more precise diagnostic results, including imaging exams undertaken with the objective of visualizing soft tissues, lymph vessels, and lymph nodes, and which constitute a method that can identify the pathophysiologic changes of lymphedema.^9,10^

Diagnostic ultrasound (DUS) is one of the new methods of lymphedema assessment for evaluating limbs with edema. It is used to detect whether the etiology is entirely venous or if there is also a lymphatic abnormality (phlebolymphedema). Using DUS offers the advantages that it is a simple imaging exam that is noninvasive and readily available for visualizing blood vessels. Although enlarged lymph nodes can very often be seen, DUS cannot provide images of the lymphatic vasculature. However, the ultrasonographic characteristics of the tissue layers in the limb with edema offer important information about the etiology of the edema, with the advantage of enabling follow-up of treatment response, measuring the thickness of each limb tissue element before and after treatment.^11^

Diagnostic ultrasound can be used to assess and diagnose lymphedema in upper limbs, lower limbs, and genital organs and can offer differential diagnosis between several different pathologies that cause increased limb volume. Moreover, DUS is a relatively inexpensive method for examining the characteristics of soft tissues and can reliably be used for lymphedema diagnosis, since it enables assessment of the thickness of the skin and subcutaneous tissue and can measure tissue compliance in clinical settings.^12,13^

Despite progress in treatment approaches for lymphedema, it remains necessary to conduct more studies to improve care. Scientific studies have demonstrated that lymphedema assessment methods lack consistency and rigor, requiring a more precise technique for diagnosis and follow-up, particularly for early detection and precise classification. Early diagnosis of lymphedema enables safe intervention, which can reverse development and enable more accurate management of treatment, since treatment depends on disease severity, making precise classification necessary.^13^

In view of the need to examine DUS as a resource for detection and monitoring of lymphedema, the objective of this study is to conduct a review of the subject, focusing on its use for measurement and examination of structural changes in affected limbs.

## METHOD

This is a systematic literature review based on database searches for articles that deal with the use of ultrasound for LSBC. The study was conducted in accordance with the recommendations of the Preferred Reporting Items for Systematic Reviews and Meta-Analysis (PRISMA) and was performed from January to December of 2022.^14^

Identification of potential studies for analysis employed a wide-ranging strategy involving cross-referencing of specific search terms. The review started by searching the contemporary literature in Brazilian and international articles indexed on databases such as PubMed, Lilacs, IBECS, MEDLINE, Cochrane Library, EMBASE, SciELO, and Google Scholar. Electronic searches were conducted using keywords such as lymphedema, ultrasound, breast cancer, and upper limb in both Portuguese and English. The descriptors employed were chosen taking into consideration their relevance for representation of the subject and their use in the specialist scientific literature. The keywords were taken from the Biblioteca Virtual em Saude (BVS), specifically the Descritores em Ciências de Saude (DECS), and from Medical Subject Headings (Mesh), and Emtree.

The objective of this review is to offer researchers contact with what has been written on this research subject, contributing to construction of knowledge about use of ultrasonography in LSBC. A descriptive, bibliographic study was conducted on the basis of scientific articles available over the internet via scientific databases. The historical review attempts to collect what has been written on the subject, improving knowledge about the subject.

The inclusion criteria defined as eligible articles published in Portuguese, English, or Spanish on the subject of the review describing cross-sectional or longitudinal studies or accuracy studies. No date limits were set because of the importance of the subject and the small number of articles published on it and also to offer a temporal description of articles published on the subject, the importance of which from a historical perspective justifies conducting the study. Literature reviews, systematic reviews, and articles that did not cover the subject were excluded.

The articles selected using the search strategies described were exported to Rayyan Qatar Computing Research Institute (Rayyan QCRI) review software for independent analysis by two researchers previously blinded for assessment of the articles. The Rayyan program was initially used to exclude duplicates, followed by screening by titles, abstracts, and full text. The analysis involved assessment of methodological quality, intervention proposals, and outcomes achieved. At the end of the analysis, any disagreements were solved through consensus between the researchers.^15^

All articles found were evaluated to identify those that directly dealt with the subject. During analysis of the articles, choices were made between thematic areas, analyzing content as well as titles, since the title is not always indicative of a study’s scope.

**Figure 1:**
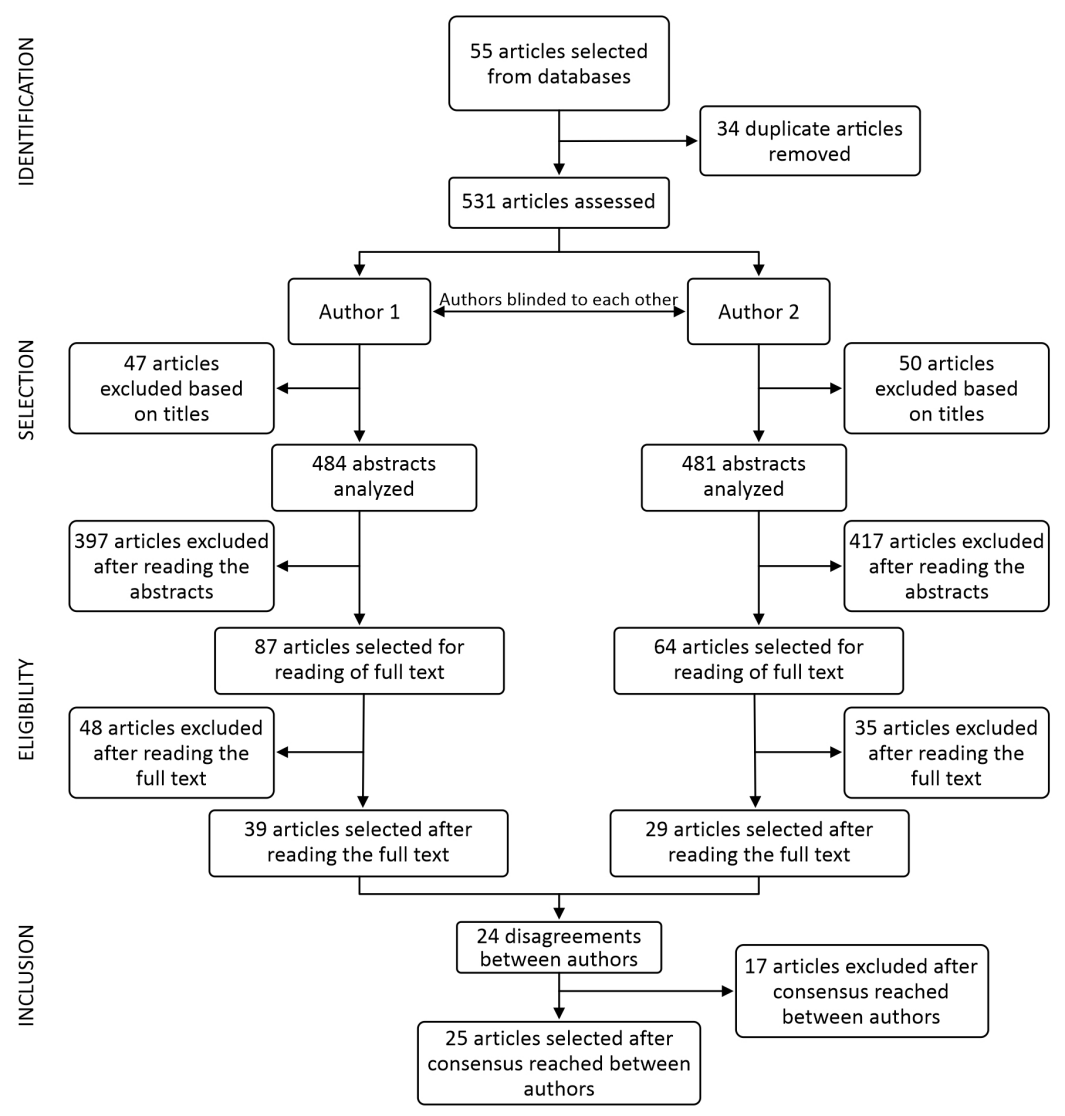
Study flow diagram.

Each article selected underwent analytical reading, with integral and interpretative analysis of the text, followed by identification of its principal concepts and synthesis of its main ideas. The data identified were then organized in a table, in which the information was organized as follows: sample size, type of study, intervention implemented, main findings, whether the intervention was effective, and equipment specifications. This cataloging process was done to facilitate understanding of the articles analyzed in the study. Figure 1 shows the research development flowchart.

## RESULTS

A total of 565 articles were selected from the databases, which, after screening and analysis, yielded 25 articles for cataloging. The results constitute the findings of evaluation of 25 articles selected by consensus between the authors. The flow diagram contained in the Methods section illustrates how these studies were selected.

The authors of all 25 articles published from 2004 to 2021 described the efficacy of DUS as a diagnostic method for lymphedema. Only one article, by Duyur Çakit et al.,^16^ discussed relative efficacy, evaluating only a non-obese population with grade 2 LSBC.

Table 1 lists the principal points discussed in the articles.

**Table 1:** Principal points discussed in the articles.

**Author (Location)**	**Sample characteristics**	**Level of evidence**	**Intervention**	**Main findings**	**Results**	**Equipment specifications**
Mellor et al.^17^ (United Kingdom)	10 women with LSBC, aged 48 to 75 years, post-axillary radiotherapy.	1c	DUS was used for the assessments. Measurements were conducted in the morning to eliminate the diurnal variation effect on water content, skin thickness, and echogenicity.	Measurement with high frequency DUS can yield a simple, reliable, and useful result for investigating lymphedema and to assess therapeutic interventions.	Effective: measurement of skin thickness with DUS is a clinical tool that is useful in the diagnosis of lymphedema, in addition to helping in investigation of therapeutic techniques.	Two systems were used in the study:
						1. Dermascan ultrasound (Dermascan C, Cortex Technology, Smedevaenget, Denmark),at 20 MHz.
			Subcutaneous thickness measurement encompassed measurements defined for 4.0 cm in width and 4.0 cm in depth.			
						2. Acuson XP10 (Acuson, Mountain View, CA, United States) with a frequency of 7 MHz
				The subcutis was considered the principal site of swelling. The results indicate that lymphedema has a considerable impact on the skin around the arm, irrespective of the exact location of the subcutis with edema.		
Han et al.^18^ (South Korea)	20 healthy individuals and 20 women with LSBC.	1c	In the healthy individuals, thickness of the UL dermis and the subcutis was measured bilaterally.	DUS was able to provide valuable information on the extent of edema and fibrosis of skin and subcutis and is a useful tool to follow the results of lymphedema treatment and its progression over time.	Effective: DUS is able to provide valuable information on the extent of edema and fibrosis of skin and	System: 12 MHz linear probe for Logiq E (GE Healthcare Ultrasound,
			In the patients with lymphedema, staging was defined using the Casley-Smith Lymphedema Staging System.			
						Milwaukee, United States)
					subcutis.	
			DUS was used to measure the thickness of the dermis and the subcutis.			
Lee et al.^9^ (Taiwan)	60 patients with lymphedema post-breast cancer surgery.	1b	Tissue thickness was measured with DUS at three points before and after CPT.	In diagnosis of lymphedema, measures of skin, subcutaneous, and total soft tissue thickness of the upper extremity were greater than for the unaffected side.	Effective: ultrasonographic assessment was effective for assessment of the results of CPT in LSBC.	Information not provided.
			Soft tissue thickness was defined as the sum of skin and subcutaneous tissue thickness.			
				The ultrasound measurements were reliable and revealed that CPT was effective for reducing the thickness of soft tissues.		
Devoogdt et al.^19^ (Belgium)	42 patients with unilateral axillary dissection for primary breast cancer took part and were assessed for evolution of lymphedema secondary to breast cancer.	1b	Ultrasound was used to investigate evolution of thickness and echogenicity of cutis and subcutis up to 1 year after axillary dissection for breast cancer and compare patients with and without objective lymphedema. Ultrasonographic assessments of both arms were conducted immediately and 6 and 12 months after axillary surgery. Sagittal and transverse images were acquired at each measurement site. The reference point was placed at the center of the probe and minimal pressure was applied.	The ultrasonographic assessment found that subcutaneous echogenicity was more frequently disturbed on the affected side (in 7-33% of the patients) than on the healthy side (0-19%). The prevalence of changed echogenicity of the subcutaneous of the affected arm (not significant) was clinically relevant and was different between patients with and without lymphedema at the wrist, dorsal forearm, and biceps and triceps. According to the study, it appears that increased subcutaneous thickness at the ventral forearm and triceps and disturbed echogenicity of the cutis at the wrist are good indicators for identifying patients with lymphedema	Effective: in patients with breast cancer, ultrasonography can be useful to diagnose lymphedema in the arm; but cannot be used as a separate diagnostic test for lymphedema.	Ultrasound (Siemens Acuson Antares Premium, Erlangen, Germany) machine with a high frequency 13 MHz linear probe.
Abreu et al.^20^ (Brazil)	80 patients post-mastectomy and radiotherapy divided into two groups: 40 patients with LSBC and 40 without the disease.	1b	Ultrasonographic abnormalities were assessed in the transverse and longitudinal aspects.	There was an overall prevalence of 83.8% of ultrasonographic abnormalities in the axillary vein of women with LSBC.	Effective: the prevalence of ultrasonographic abnormalities was greater among patients with LSBC.	Ultrasonography system model Sonoace X8 or SA 8000EX Prime, with 5-12 MHz multifrequency linear transducer, both by Medison Co. Ltd., 1003 Daechi-dong, Gangnam-gu, Seoul 135-280 South Korea.
Bok et al.^5^ (South Korea)	32 patients with lymphedema secondary to breast cancer were randomized: one group received CPT + PRE and the other group PRE only.	1b	The thickness of subcutaneous tissue and muscle was measured with DUS.	DUS is a good tool for measuring changes in muscle thickness after PRE to confirm the effect of lymphedema treatment. It can be used to diagnose lymphedema and as a method to determine treatment.	Effective: it was possible to use DUS to assess the effects of PRE in treatment of patients with LSBC.	Ultrasound machine (MyLab 50, Esaote, Italy).
						Information on system frequency not provided.
			Measurements taken at two points: 1) proximal upper limb, 10 cm proximal of the tip of the elbow; and 2) distal upper limb, 10 cm distal of the tip of the elbow.			
Johnson et al.^21^ (United States)	17 women with lymphedema secondary to breast cancer.	1b	Ultrasound was used at two sites on all subjects’ involved and uninvolved upper extremities. 55 measures were taken for each site.	The DUS images were reliable for measurement of mean entropy between involved and uninvolved extremities at the anterior forearm. Compared with clinical edema assessment, DUS demonstrated good correlation for entropy at the inferior posterior arm.	Effective: DUS as a tool for quantifying subcutaneous tissues is a safe, mobile, and effective method for measuring the texture of lymphedematous tissues.	Sonosite M-Turbo ultrasound system with 15 MHz linear transducer.
Suehiro et al.^22^ (Japan)	30 patients with unilateral stage II breast cancer-related lymphedema of the arm took part in the study.	1b	Ultrasonography was used to investigate skin thickness, SELEB, and SCT, and the degree of increase in SEG and SEFS in arms with lymphedema. Skin and subcutaneous tissue of both arms of 30 patients with unilateral stage II lymphedema secondary to breast cancer were examined at five points (medial/lateral upper arm and forearm and back of the hand). The degrees of SEG and SEFS were determined according to severity (interval: 0-2).	All of the parameters measured, except SEFS in the medial arm, were significantly higher on the side with lymphedema than on the normal side. Parameters differed most noticeably at the medial forearm. It was not possible to confirm an increase in SEG/SEFS scores according to severity, i.e., higher SEG/SEFS scores in the forearm than in the arm.	Effective: ultrasound showed good capacity to demonstrate skin thickness, SELEB and SCT, and SEG and SEFS grades in arms with lymphedema and normal arms in patients with LSBC and also showed that increases in these parameters were greater in the medial forearm of the arm with lymphedema.	Ultrasound system (LOGIQ S6; GE Healthcare, Little Chalfont, Buckinghamshire, United Kingdom) with a 7 to 12 MHz linear transducer.
Dai et al.^23^ (Japan)	10 UL with LSBC with a history of ADLA and 14 UL with LSBC.	1c	Asymmetry was calculated by histogram analysis for ROI defined in images of the dermis, using the same technique for both limbs.	Distribution of collagen in the papillary layer of the dermis was different after ADLA episodes, based on the results for asymmetry and elevated pixel echogenicity.	Effective: DUS is effective for identification of structural changes in ADLA, a risk factor for increased lymphedema.	Ultrasound Derma scan C (Cortex Technology, Smedevaenget, Denmark) at 20 MHz.
				DUS is useful for assessing asymmetry and confirming dermal structure.		
Hansdorfer-korzon et al.^24^ (Poland)	35 women with LSBC were enrolled and 29 completed the study.	1b	Ultrasonography (B-mode) was used to assess lymphedema in the side of the chest after mastectomy. This test was performed three times at a specific site on the operated side and symmetrically on the opposite side. Subsequently, patients were fit with an appropriate compression corset and reassessed with ultrasonography.	Ultrasonography identified subcutaneous changes caused by lymphedema.	Effective: ultrasound was effective for assessment of the effects of the proposed treatment.	Ultrasound (Voluson E8, ML6-15 probe; GE Healthcare, Piscataway NJ, United States). Transducer and frequency were not reported.
Jeon et al.^3^ (South Korea)	32 patients with LSBC randomized into 2 groups: PRE and no PRE.	1b	Thickness of muscle and subcutaneous tissue were measured with DUS. Muscle and subcutaneous tissue thicknesses were measured at baseline and at 4 weeks and 8 weeks after PRE.	Initial muscle thickness of all participants was significantly smaller in the lymphedematous arm compared with the unaffected UL. The subcutaneous tissue was thicker in the UL with LSBC.	Effective: DUS is one of the best tools for diagnosis and to determine efficacy of treatment for LSBC.	Information not provided.
Yang et al.^13^ (United States)	Clinical feasibility was tested with four participants: two patients with LSBC and two healthy volunteers.	1c	2D deformation imaging method using registration of pre- and post-compression ultrasound B-mode images The method was tested through a series of experiments using elastography under various pressures.	The initial findings are encouraging and a large clinical study is needed to further evaluate this 2D ultrasound strain imaging technology.	Effective: DUS 2D was effective for identification of UL changes caused by lymphedema.	Clinical scanner (SonixTouch, Ultrasonix, British Columbia, Canada) with a linear matrix transducer (L14-5 / 38). 10 MHz central frequency.
Hashemi et al.^25^ (Canada)	7 women with stage 2 LSBC were assessed.	1b	DUS identified the properties of tissues in women with LSBC.	Ultrasonographic elastography assessment was effective for staging LSBC and assessing tissues.	Effective: new DUS elastography techniques can be used to better evaluate o LSBC and provide treatments to reduce progression of the condition.	Ultrasound system: Alpinion E-Cube (Bothell, WA, United States) using an L3-8 transducer with a central frequency of 10 MHz and sampling rate of 40 MHz.
			Quasi-static ultrasound elastography techniques were used to investigate their usefulness in staging lymphedema.			
Yang et al.^26^ (South Korea)	158 women at least 6 months after treatment for unilateral breast cancer with or without lymphedema were recruited retrospectively.	1b	DUS was used to assess subcutaneous echogenicity of the medial arm and forearm on both sides and graded by subcutaneous echogenicity grade.	DUS is indicated for assessment of lymphedema, primarily in the medial forearm.	Effective: ultrasound subcutaneous echogenicity can improve the precision of diagnosis of lymphedema of the forearm.	Ultrasound equipped with a 11 MHz transducer.
						The system used was not reported.
Iyigun et al.^27^ (Turkey)	36 female patients with stage 1 or 2 lymphedema of upper limbs secondary to breast cancer.	1b	Ultrasonography was used to make a total of three measurements of the arm with lymphedema and the normal extremity, one 10 cm proximal of the styloid apophysis of the ulna, for the forearm, and 10 cm proximal of the medial epicondyle, for the arm. Images were acquired of 10 different subcutaneous regions and used to calculate the mean shear wave velocities.	The shear wave elastography ultrasound technique was able to identify areas with lymphedema.	Effective: ultrasonography is a useful tool for distinction and diagnosis of initial and late stages of lymphedema.	SWE ultrasound (Acuson S 3000 US) 9L4 transducer with frequency range of 4-9 MHz.
Mander et al.^28^ (United States/Italy)	287 women with LSBC.	1a	Tissue thickness was measured and compared considering the contralateral limb as the control. The limb was considered affected by lymphedema if there were two consecutive circumference measurements more than 2 cm larger than the contralateral limb.	Traditional DUS can provide secondary upper limb lymphedema characterization with related mapping and useful data for better lymphatic physiopathology understanding and for a properly addressed therapeutic protocol.	Effective: DUS proved effective for characterization of LSBC.	Ultrasound (Sono Scape S22, linear probe 12L-A, 192 elements, 6-16 MHz).
Polat et al.^29^ (Turkey)	41 women with a history of unilateral breast surgery and axillary dissection or excision of sentinel lymph nodes.	1b	The thickness and stiffness of cutaneous and subcutaneous tissues of the forearm and arm were measured with ultrasound and SWE. The affected limb was compared with the contralateral limb.	In the latent lymphedema group, the thickness measurements of the cutaneous tissue of the affected forearm and the cutaneous and subcutaneous tissue of the affected arm were significant.	Effective: DUS was effective for diagnose of LSBC even at a latent stage.	B-mode ultrasound -Acuson S2000 US system (Siemens Medical Solutions, Mountain View, CA, United States) equipped with a 9 MHz probe
Suehiro et al.^30^ (Japan)	120 patients who had undergone surgery for breast cancer and were monitored for emergence of lymphedema.	1b	Ultrasonography of skin and subcutaneous tissue was used to assess the echogenicity of the limbs assessed with the objective of determining its diagnostic capacity for early detection of post-mastectomy lymphedema from 1 preoperative month up to 2 years during the postoperative period. Assessment of diffuse increases in echogenicity in the subcutaneous layer and echogenic lines.	Ultrasound found evidence of differences in subcutaneous echogenicity between the regions assessed in the upper limbs assessed for development of lymphedema secondary to breast cancer.	Effective: ultrasonography was able to identify areas with increased cellular density and increased tissue collagen content, which indicates presence of subcutaneous inflammation, which shows presence of lymphedema.	Ultrasound System Logiq S6 (GE Healthcare, Little Chalfont, Buckinghamshire, United Kingdom) with a 7 to 12 MHz linear transducer.
Giray and Yağcı^8^ (Turkey)	50 women with breast cancer-related lymphedema of the arm.	1b	Ultrasound was used to assess interrater and intrarater reliability for diagnosis of lymphedema by identification of degree of subcutaneous echogenicity and the degree of lymphedema secondary to breast cancer, which enables semiquantification of nonspecific inflammation of the subcutaneous tissue and fluid accumulation in lymphedema secondary to breast cancer. The probe was maintained in an axial position on the medial forearm over the flexor carpi radialis muscle. The depth of image acquisition was set at 2 cm.	Ultrasonography showed that SEG grade and SEFS grade are both reliable according to intraexaminer and interexaminer assessments, but it should be considered that examiners had lower agreement when classifying SEG in patients at intermediate clinical stages and higher agreement when classifying SEFS grade in patients at intermediate clinical stages.	Effective: based on the findings of this study, SEG and SEFS demonstrated acceptable reliability. The ultrasound SEG and SEFS classification system can be useful for monitoring progression, composition, and management of lymphedema secondary to breast cancer.	Esaote MyLab ultrasound system with 6-18 MHz linear matrix probe.
Hashemi et al.^31^ (Canada)	The study population comprised 7 women with stage 2 breast cancer-related lymphedema.	1c	Ultrasound elastography was used to compare the mechanical properties of the affected and unaffected arms to offer an alternative to current subjective assessment of lymphedema secondary to breast cancer. The method was compared to the pitting test habitually used to assess lymphedema. Ultrasound data were collected from both arms of seven patients with stage 2 lymphedema, at six different locations in each arm to identify changes to the mechanical properties of tissues related to detection and staging of lymphedema.	Ultrasound elastography was able to visualize differences in the tissue properties of the unaffected limb (not lymphedematous) and the affected limb (lymphedematous). The values for deformation rate in the affected limb are consistent and significantly lower in skin than in subcutaneous fat and skeletal muscle layers. Lower deformation rates were observed in affected skin compared with the unaffected limb.	Effective: the elastography technique proposed is more sensitive than the pitting test.	Ultrasound system Alpinion E-Cube (Bothell, WA, United States) using an L3-12H transducer with a central frequency of 10 MHz and sampling rate of 40 MHz.
Seo et al.^7^ (South Korea)	6 women who had undergone surgery for breast cancer and were diagnosed with unilateral upper limb lymphedema.	1c	Ultrasonography was used to assess the effects of an intervention with MLD, pre-intervention and post-intervention. Limb volume measurements were performed of the affected and contralateral limbs, which were compared.	Ultrasound images showed significant differences in the volume of the affected limb compared to the unaffected side. On the affected side, although ultrasonography showed a significant reduction after MLD, there was no significant difference when compared to baseline.	Effective: ultrasonography proved effective for assessment of the treatment approach employed (MLD).	Ultrasound (MySono U5; Samsung Medison Co., Seul, South Korea) with a 7.5 MHz linear transducer.
Niwa et al.^32^ (Japan)	The study enrolled 20 women who had been treated for unilateral breast cancer and later developed upper limb lymphedema.	1b	Subcutaneous tissue was scanned with an ultrasound system using a 6 to 15 MHz linear transducer to assess the capacity of tissue texture characteristics to discriminate the presence of accumulated fluid within the subcutaneous tissue of breast cancer-related lymphedema. Fluid accumulation was observed using a 3-Tesla MR system under double-echo steady-state conditions.	There was a significant difference in textural features among the three groups (with hyperintense area, without hyperintense area, and unaffected side), revealing significant differences in seven textural features within the hyperintense area, showing it was possible to discriminate presence of fluid accumulation in subcutaneous tissue of LSBC with ultrasound images.	Effective: the study showed that seven textural features quantified with US imaging data can provide information on fluid accumulation in subcutaneous tissue with lymphedema.	Ultrasound: Sonosite Edge II; Sonosite, Inc., FUJIFILM) using a 6 to 15 MHz linear transducer.
Kim et al.^33^ (South Korea)	69 female patients with a diagnosis of stage 1 lymphedema secondary to advanced breast cancer.	1b	Ultrasonography was performed on both arms of each subject, with the patients lying down. The examiner marked the regions to measure a cross-sectional area.	The cross-sectional area measurement method showed high coefficients for lymphedema assessment. Stiffness of soft tissues, which reflects their histological status, can be measured and reveal different characteristics to tissues with the same volume and lymphedema.	Effective: a combination of these two ultrasonographic methods appears to show not only structural changes but also histological changes in soft tissues after development of lymphedema.	Ultrasound (LOGIQ E9; General Electric, Boston, MA, United States) with a 7.5 MHz transducer.
			Subcutaneous tissue stiffness was also obtained by measuring thickness differences of soft tissue when applying minimal and maximal pressure to the skin (compliance) and its ratio to the initial thickness.			
Erdinç Gündüz et al.^34^ (Turkey)	34 female patients with lymphedema secondary to breast cancer. Unilateral breast cancer-related lymphedema.	1b	Skin and subcutaneous thicknesses were measured ultrasonographically from four quadrants at the marked points and also subcutaneous tissue changes were graded according to the subcutaneous echogenicity grade (SEG) scale ultrasonographically. Ultrasonographic measures performed were subcutaneous ultrasonographic echogenicity and skin and subcutaneous thickness measurement.	Lymphedema severity was graded ultrasonographically according to the SEG scale as stage 0, stage 1, and stage 2, assessing the echogenic lines of echogenicity. Ultrasonographic assessment of the difference between the two upper extremities had a high (0.83%) sensitivity and an acceptable (0.75%) specificity in the differentiation of Grade II and Grade III lymphedema.	Effective: a correlation was established between circumferential measurements and ultrasonographic measurements. Ultrasonography can be used complementary to circumferential measurements in diagnosing lymphedema.	Information not provided.
Duyur Çakit et al.^16^ (Turkey)	The study enrolled 47 patients with unilateral upper limb lymphedema secondary to breast cancer.	1b	Ultrasound was conducted to determine its role in the follow-up of effectiveness of CDT in different subgroups of patients with breast cancer-related lymphedema. All patients underwent CDT, the circumference measurements and ultrasonographic soft tissue thicknesses evaluations were performed at two anatomic sites, and upper extremity limb volumes were calculated using the truncated cone formula before and after CDT.	There were significant decreases in both circumferential measurements and ultrasonographic soft tissue thicknesses in non-obese patients and stage 2 lymphedema patients after 15 sessions of CDT. The ultrasonographic soft tissue thickness values were correlated with the upper arm and forearm circumference values before and after CDT.	Relative efficacy: ultrasonography is a reliable method to measure the soft tissue thickness and treatment efficacy after CDT in non-obese and stage 2 patients with LSBC only.	Ultrasound system with 7-12 MHz linear transducer: Logic P5, GE medical systems, Wisconsin, United States.

UL = upper limb; DUS = diagnostic ultrasonography; LSBC = lymphedema secondary to breast cancer; CPT = complex physiotherapy; PRE = progressive resistance exercise; ADLA = acute dermatolymphangioadenitis; MLD = manual lymph drainage; SELEB = subepidermal low echogenic band; SCT = subcutaneous tissue; SEG = subcutaneous echogenicity; SEFS = subcutaneous echo-free space; ROI = region of interest; CDT = complex decongestive therapy; SWE = shear wave elastography.

Polat et al.^29^ and Iyigun et al.^27^ reported on possible time of diagnosis: considering DUS feasible in the latent stage, initial stages, and late stages. Additionally, Yang et al.^26^ correlated echogenicity of ultrasonographic waves as a method for improving diagnosis of lymphedema.

Duyur Çakit et al.^16^ discussed use of DUS for monitoring the efficacy of complex decongestive therapy in different subgroups and suggested the instrument’s relative efficacy, showing that non-obese and stage 2 patients with LSBC can be assessed with greater reliability.

The process of lymphedema formation involves increased activity of neutrophils, macrophages, and fibroblasts and inflammation and collagen deposition (fibrosis). According to Suehiro et al.,^30^ DUS can be used to capture the increase in collagen and the increase in subcutaneous inflammation, while Kim et al.^33^ showed that it is also possible to detect histological changes in addition to structural ones. Finally, Han et al.^18^ describe fibrosis as a highly valuable ultrasonographic finding, in addition to the extent of edema.

Seven of the 25 selected articles from 2004 to 2022 considered use of DUS in conjunction with therapeutic techniques with the objective of demonstrating the progress or regression obtained during treatment. Seo et al.^7^ used manual lymph drainage (MLD) as lymphedema treatment method and reported the efficacy DUS during the process. Devoogdt et al.^19^ suggested that another diagnostic method in conjunction is needed until there is more scientific evidence.

Elastography conducted using DUS is a method that can identify tissue rigidity, observing the presence of possible nodules, and is painless and minimally invasive. Four authors demonstrated the efficacy of elastography. Hashemi et al.^25^ showed the importance of the method not only for diagnosis, but also for staging LSBC. Hashemi et al.^31^ described elastography as more sensitive than the pitting test.

The ultrasound systems employed varied in terms of model and frequency. Mellor et al.^17^ and Dai et al.^23^ used a Dermascan (20 MHz) system. Four articles did not specify what equipment was used and Bok et al.^5^ and Hansdorfer-Korzon et al.^24^ did not report the frequency employed. Han et al.^18^ and Suehiro et al.^22^ used a Logiq model. None of the other instruments and frequencies coincided.

Patients and studies varied in many different ways and no pattern emerged. There were 11 cross-sectional studies, two cross-sectional accuracy studies, and one descriptive cross-sectional study, while there were 11 longitudinal prospective studies and just one longitudinal retrospective study. Han et al.^18^ and Abreu et al.^20^ separated participants into two groups, the first comprising patients with LSBC and the second containing healthy patients, but they differed in terms of the number of patients in each group.

**Figure 2:**
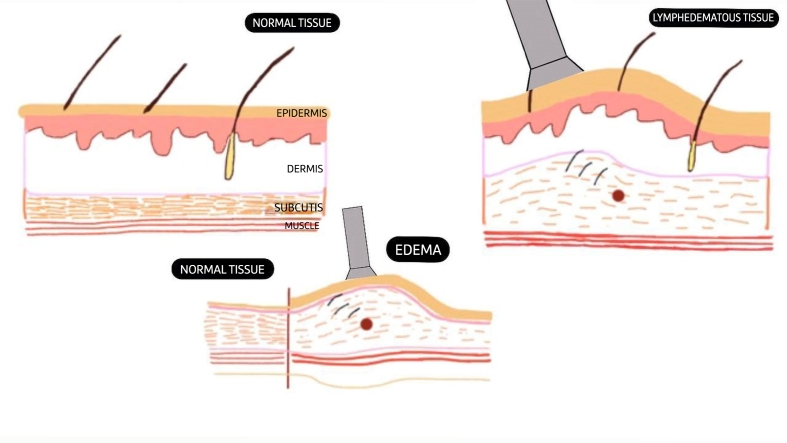
Diagram illustrating normal tissue and lymphedematous tissue.

Figure 2 shows a schematic illustration of the difference between normal tissue and tissue with LSBC assessed by DUS.

Table 1 shows the evidence level of each article. This systematic review included experimental articles, the majority with 1b and 1c evidence levels, according to the Oxford Scale for levels of evidence. The majority of evidence available is level 1, and the procedure is recommended (recommendation grade A).

## DISCUSSION

This study conducted a wide-ranging review of 25 scientific articles that documented use of DUS as a diagnostic method in lymphedema cases. There was consensus on the instrument’s efficacy for identifying edema in subcutaneous tissue.

Currently there is no specific tool for diagnosis in the initial stages, when symptoms have not yet emerged. The most popular tests for characterizing LSBC include the following: arm circumference measurement; Perometry, which assesses the volume of the affected arm compared with the unaffected arm; and bioimpedance, which tests resistance to painless electric currents passed through the arm.^35^ Other imaging methods used to assess LSBC are computed tomography, magnetic resonance imaging, and indocyanine green lymphography; but they are not portable and they are more expensive.^8^ On the other hand, the lymphoscintigraphy imaging technique is considered the standard criterion for diagnosis of LSBC, using a radiotracer to show the lymphatic system and reveal the presence and caliber of the lymph vessels, lymph nodes, collaterals, and delayed radiotracer uptake. However, this method is not generally preferred because of the lack of a standard protocol, the invasivity of the procedure, and patient exposure to radiation.^35^

Ultrasonography is a safe, easy, and inexpensive procedure for assessment of patients with LSBC. Changes include increased thickness of the dermis, changes from hypoechogenicity to hyperechogenicity in subcutaneous tissue, and fluid accumulation in the dermis, the interlobular space, and the superficial fascia. While these images can be difficult to detect in ultrasound images, they can provide a quantitative measurement of the thickness of cutaneous, fascial, and surrounding tissues for assessment of LSBC.^35^

Diagnostic ultrasound can also be useful as an effective prognostic tool, since it can identify patients at risk of developing an incomplete pathological response. Use of this imaging technique can reduce the time spent undergoing several invasive diagnostic procedures and can also reduce the health care costs involved in the process.^36^ Morphological and functional parameters detected using DUS can be correlated with diagnosis, staging, prognosis, and clinical therapeutic efficacy in LSBC.^37^

Ultrasound is considered a simple and safe imaging exam for assessing the thickness of the skin and subcutaneous tissue and, because of this, has been studied for assessment of patients with lymphedema. In recent years, ultrasound elastography has been used to assess LSBC, but the parameters for evaluation, diagnosis, and staging of the disease are not yet well-defined.

Diagnostic ultrasound appears to be a method that offers advantages because it is safe, minimally invasive, practical, and inexpensive, it doesn’t use ionizing radiation, it can be used preoperatively, intraoperatively, and postoperatively, and it is useful for assessing the efficacy of lymphedema treatment. The disadvantages observed were the need for a skilled operator to perform the procedure, application of the correct pressure when conducting DUS, and the need for more studies of the subject.

Finally, in clinical practice, DUS appears to be a promising resource for objective measurement, classification, and follow-up of LSBC. The procedure is rapid, painless, practical, and minimally invasive for the patients and the equipment can be found in many medical care settings.

## CONCLUSIONS

In view of the facts presented, it is understood that ultrasonography is a necessary instrument for assessment of cases of lymphedema secondary to breast cancer, since it has been shown to be effective in a more objective manner and is a resource that is feasible for diagnosis.
