# Supplementary material for: Ultrasonography as an instrument to evaluate lymphedema secondary to breast cancer: systematic review
Source: J Vasc Bras. 2023 Dec 18;22:e20220144. doi: 10.1590/1677-5449.202201441 (PMC10755892; doi:10.1590/1677-5449.202201441)
Supplement: Translation

# Translation: Ultrassonografia como instrumento de avaliação do linfedema secundário ao câncer de mama: revisão sistemática

**DOI:** 10.1590/1677-5449.202201442

**Authors:** Laura Ferreira de Rezende, João Paulo Martins Piloni, Vitória Livorato Kempa, Júlia Franco Ramos Silva, Vanessa Fonseca Vilas Boas, Regiane Luz Carvalho, Ângela Gonçalves Marx

**Affiliations:** 1 Centro Universitário das Faculdades Associadas de Ensino - FAE, São João da Boa Vista, SP, Brasil.; 2 Clínica Ângela Marx, São Paulo, SP, Brasil.

**Keywords:** ultrassonografia, linfedema relacionado a câncer de mama, neoplasias da mama

## Abstract

O linfedema é uma doença crônica e progressiva caracterizada pelo acúmulo de fluidos, provocando edema tecidual em decorrência de um sistema linfático comprometido. A ultrassonografia diagnóstica (USD) é um método capaz de avaliar as características dos tecidos moles, podendo ser utilizada de maneira confiável para o diagnóstico do linfedema, além de mensurar a complacência tecidual em um cenário clínico. Esta é uma revisão sistemática, objetivando avaliar artigos que fizessem o uso da USD na abordagem do linfedema secundário ao câncer de mama. Foram selecionados 565 artigos, que foram exportados para o programa de revisão *Rayyan QCRI* e, em seguida, triados por dois pesquisadores. Dessa busca, foram obtidos 25 artigos selecionados após consenso entre os autores e que foram catalogados quanto aos seus resultados principais. A USD foi identificada como um método vantajoso por ser seguro, pouco invasivo, de baixo custo, sem uso de radiação, além de ser útil para avaliar a eficácia de terapias no tratamento do linfedema.

## INTRODUÇÃO

O linfedema é uma grave doença crônica e progressiva caracterizada por uma alta concentração de fluidos com proteínas dentro do interstício, causado por uma obstrução parcial ou total da drenagem linfática, provocando edema tecidual em decorrência de um sistema linfático comprometido. Há um maior envolvimento do sistema linfático subfascial do que no sistema linfático epifascial à medida que o linfedema vai se tornando mais severo. Enquanto o linfedema progride, fibrócitos e/ou adipócitos iniciam a proliferação nas áreas afetadas, acarretando modificações estruturais no tecido da pele e do tecido subcutâneo, o que aumenta a vulnerabilidade a infecções bacterianas e fúngicas^1-3^ .

Entre as formas de manifestação do linfedema, destaca-se a ocorrência do linfedema secundário ao câncer de mama (LSCM). Estudos relatam que, em estágios iniciais, o linfedema apresenta-se clinicamente como edema em membro superior em região do braço, ombro, pescoço ou tronco homolateral ao tratamento, consequência da remoção de linfonodos e de vasos linfáticos regionais, causando o comprometimento do sistema linfático local e da drenagem linfática. Pode haver um aumento proteico na região tecidual afetada, resultando em inflamação crônica, fibrose, dor, limitação da amplitude de movimento ou parestesia, assim como em diminuição da função imunológica, o que aumenta o risco de inflamações e infecções locais^4-7^ .

Houve uma demonstração de que a progressão do linfedema ocorre na forma de um ciclo vicioso, no qual a estase linfática estimula o desenvolvimento da inflamação crônica, com envolvimento de uma resposta de macrófagos e células CD4 + descontrolada, acúmulo de gordura, que também propicia a ocorrência de inflamação crônica por infiltração e ativação de macrófagos, produzindo citocinas inflamatórias que, por sua vez, promovem mais estase linfática, diminuem o bombeamento linfático e aumentam a filtração capilar^8^ .

Com o objetivo de oferecer qualidade de vida, diminuindo o desconforto físico e psicológico dos pacientes, é essencial que se faça um diagnóstico preciso do problema a fim de que se tenha um melhor prognóstico e planejamento terapêutico. Apontamentos mostram que esse diagnóstico nem sempre é fácil, sendo necessário diferenciá-lo de outras patologias que apresentam condições semelhantes às apresentadas no linfedema, como edema local e fibrose dos tecidos subcutâneos. Existem muitos testes que podem ser utilizados com o objetivo de se chegar a resultados diagnósticos mais precisos, entre eles, os exames de imagem, com o objetivo de visualização de tecidos moles, vasos linfáticos e linfonodos, sendo um método que possibilita a identificação de alterações fisiopatológicas do linfedema^9,10^ .

Considerando novas modalidades de avaliação de linfedema para a avaliação de um membro edematoso, o uso da ultrassonografia diagnóstica (USD) tem por finalidade a detecção de uma etiologia venosa isolada ou em combinação com uma anormalidade linfática (flebolinfedema). O uso da USD possui como vantagem ser um teste de imagem simples, não invasivo e prontamente disponível para a visualização da vasculatura sanguínea. Embora, muitas vezes, os linfonodos aumentados possam ser visualizados, a USD não pode gerar imagens da vasculatura linfática. Entretanto, as características ultrassonográficas das camadas de tecido do membro edematoso fornecem informações importantes sobre a etiologia do edema, com a vantagem da possibilidade do acompanhamento da resposta à terapia, determinando a espessura de cada um dos elementos do tecido no membro antes e depois do tratamento^11^ .

O uso da USD possibilita a avaliação e o diagnóstico do linfedema em membros superiores, inferiores e em órgãos genitais, sendo capaz de oferecer diagnóstico diferencial entre várias patologias que levam ao aumento do volume do membro afetado. A USD é um método relativamente barato para observar as características dos tecidos moles, podendo ser utilizada de maneira confiável para o diagnóstico do linfedema, uma vez que possibilita a avaliação da espessura da pele e do tecido subcutâneo, além de mensurar a complacência tecidual em um cenário clínico^12,13^ .

Apesar da evolução na abordagem terapêutica ao linfedema, ainda é necessário o desenvolvimento de pesquisas para melhorar a assistência. Estudos científicos têm demonstrado falta de consistência e rigor nos métodos de avaliação do linfedema, necessitando de uma técnica mais precisa de diagnóstico e acompanhamento, particularmente para a detecção precoce e classificação precisa. O diagnóstico precoce do linfedema possibilita uma intervenção segura, podendo reverter seu desenvolvimento e permitindo, também, uma condução mais acurada do tratamento, visto que sua condução depende da gravidade da doença, o que torna crítica a necessidade de classificação precisa^13^ .

Diante da necessidade de se abordar o uso da USD como recurso na detecção e acompanhamento do linfedema, este trabalho tem como objetivo realizar uma revisão sobre o assunto, focando seu uso na medição e nas alterações estruturais em membros afetados.

## MÉTODO

Este trabalho trata-se de uma revisão sistemática de literatura, cuja busca se baseou em bases de dados, avaliando artigos que abordassem o uso do ultrassom na abordagem do LSCM. Esta pesquisa foi elaborada em conformidade com as recomendações do *Preferred Reporting Items for Systematic Reviews and Meta-Analysis* (PRISMA) e realizada no período de janeiro a dezembro de 2022^14^ .

A identificação dos potenciais estudos para avaliação foi realizada através de estratégia abrangente, utilizando o cruzamento dos descritores definidos. A revisão se baseou na busca de literatura contemporânea em artigos nacionais e internacionais disponíveis e acessados em bases de dados como PubMed, Lilacs, IBECS, MEDLINE, Biblioteca Cochrane, EMBASE, SciELO e Google Scholar. Para a pesquisa em meio eletrônico, foram utilizados descritores como linfedema, ultrassom, câncer de mama e membro superior, sendo usados também na busca em bases de dados internacionais os seus correlatos em inglês. Os descritores utilizados foram escolhidos levando em consideração a sua relevância na representação do tema proposto e por serem utilizados em literatura científica especializada, sendo extraídos da Biblioteca Virtual em Saúde (BVS), mais precisamente nos Descritores em Ciências de Saúde (DECS). Foram também definidos com base no *Medical Subject Headings* (Mesh) e Emtree para delimitação de palavras-chave que seriam utilizadas na busca nas bases de dados.

Esta revisão teve como objetivo possibilitar aos pesquisadores o contato com o que foi abordado sobre o assunto da pesquisa, contribuindo para a construção do conhecimento a respeito da utilização da ultrassonografia na abordagem do LSCM. Foi realizada pesquisa bibliográfica com caráter descritivo, elaborada a partir de artigos científicos disponibilizados pela internet em bancos de dados científicos. A revisão histórica buscou recuperar o que já foi escrito sobre o assunto, permitindo aprimorar os conhecimentos sobre o tema.

Como critérios de inclusão, foram considerados elegíveis artigos em português, em inglês ou espanhol que abordassem o assunto deste trabalho e que se tratassem de pesquisas transversais, longitudinais ou de acurácia. Não foi delimitado um período limite para artigos em razão da importância do assunto e da pequena quantidade de artigos publicados sobre esse assunto, assim como para fazer um acompanhamento temporal de artigos publicados sobre o tema abordado, cuja importância na retratação histórica motivou este trabalho. Foram excluídos artigos de revisões de literatura, revisões sistemáticas e aqueles que não fizeram abordagem do tema referido.

Os artigos selecionados através das estratégias de pesquisa previamente descritas foram exportados para o programa de revisão *Rayyan Qatar Computing Research Institute* (*Rayyan QCRI*), no qual foram analisados por dois pesquisadores independentes previamente cegados na avaliação dos artigos. No programa *Rayyan*, inicialmente, foram excluídas todas as duplicatas, seguindo para a triagem pela avaliação de títulos, resumos e textos completos. A análise se baseou na avaliação da qualidade metodológica, na proposta de intervenção e nos desfechos alcançados. Ao final da análise, os conflitos foram solucionados através de consenso entre os pesquisadores^15^ .

Todos os artigos encontrados foram avaliados a fim de buscar aqueles que abordaram diretamente o tema desejado. Para a análise dos artigos utilizados, a escolha se deu entre as áreas temáticas, sendo analisado, além dos títulos, o conteúdo, uma vez que o título nem sempre é indicativo da abrangência do trabalho.

Cada artigo selecionado passou por leitura analítica, com análise integral e interpretativa do texto, seguida da identificação dos conceitos principais e da sintetização das ideias principais. Após esse processo, os dados encontrados foram organizados em uma tabela, na qual as informações foram organizadas conforme: o tamanho da amostra, o tipo de estudo, a intervenção realizada, os principais achados, se a intervenção foi eficaz e a especificação do equipamento. Essa catalogação foi realizada a fim de facilitar a compreensão dos artigos abordados na pesquisa. A Figura 1 apresenta o fluxograma de desenvolvimento da pesquisa.

**Figura 1:** Fluxograma de desenvolvimento da pesquisa.

## RESULTADOS

Foram selecionados, nas bases de dados, 565 artigos que, após triagem e análise, resultaram em 25 artigos escolhidos para catalogação. Os resultados equivalem ao avaliado em 25 artigos selecionados após consenso entre os autores. O fluxograma, contido nos métodos demonstra a forma de seleção dos trabalhos.

Entre os 25 artigos relatados de 2004 a 2021, todos os autores descreveram a eficácia da USD como método diagnóstico de linfedema. Apenas um artigo, referente aos autores Duyur Çakit et al.^16^ , discorreu sobre a eficácia relativa, por ter avaliado apenas população não obesa com LSCM grau 2.

A Tabela 1 expõe todos os pontos principais discutidos nos artigos.

**Tabela 1:** Pontos principais discutidos nos artigos.

**Autor (Local)**	**Característica da amostra**	**Grau de evidência**	**Intervenção**	**Principais achados**	**Resultados**	**Especificação do equipamento**
Mellor et al.^17^ (Reino Unido)	10 mulheres com LSCM, entre 48 a 75 anos, pós-radioterapia axilar.	1c	A USD foi utilizada para a avaliação. As medidas foram feitas na manhã para eliminar o efeito da variação diurna no conteúdo de água, na espessura da pele e na ecogenicidade.	A medição com USD de alta frequência pode apresentar um resultado simples, confiável e útil para o linfedema e para investigar intervenções terapêuticas.	Eficaz: a medida da espessura da pele com USD é uma ferramenta clínica útil no diagnóstico de linfedema, além de auxiliar na investigação de técnicas terapêuticas.	Foram usados dois equipamentos no estudo:
						1. Ultrassom Dermascan (Dermascan C, Cortex Technology, Smedevaenget, Dinamarca) a 20 MHz.
				O subcutâneo foi considerado o principal local do inchaço. Os resultados indicam que o linfedema afeta muito a pele ao redor do braço, independentemente da posição exata do subcutâneo com edema.		
			A medida da espessura do subcutâneo abrangeu as medições definidas para 4,0 cm de largura e 4,0 cm de profundidade.			
						2. Acuson XP10 (Acuson, Mountain View, CA, EUA) com uma frequência de 7 MHz
Han et al.^18^ (Coreia do Sul)	20 indivíduos saudáveis e 20 mulheres com LSCM.	1c	Nos indivíduos saudáveis, foram medidas a espessura da derme e do subcutâneo dos MMSS, bilateralmente.	A USD foi capaz de oferecer informações valiosas sobre a extensão do edema e fibrose da pele e subcutâneo, sendo uma ferramenta útil para acompanhar os resultados do tratamento do linfedema e sua progressão ao longo do tempo.	Eficaz: a USD é capaz de fornecer informações valiosas sobre a extensão do edema e fibrose da pele e subcutâneo.	Equipamento: sonda linear de 12 MHz de frequência para o Logiq E (GE Healthcare Ultrasound, Milwaukee, EUA)
			Nos pacientes com linfedema, o estágio foi definido a partir do Sistema de estadiamento de linfedema de Casley-Smith.			
			Com a USD, foi medida a espessura da derme e do subcutâneo.			
Lee et al.^9^ (Taiwan)	60 pacientes com linfedema pós-cirurgia por câncer de mama.	1b	A espessura do tecido foi medida pela USD em três pontos antes e depois da TFC.	No diagnóstico do linfedema, as medidas de espessura da pele, subcutâneo e total de tecidos moles da extremidade superior afetada foram maiores do que aquelas do lado não afetado.	Eficaz: a avaliação ultrassonográfica foi eficaz na avaliação dos efeitos da TFC em LSCM.	Não informado.
			A espessura do tecido mole foi definida como a soma da espessura da pele e do tecido subcutâneo.			
				As medidas ultrassonográficas colhidas foram fidedignas e revelaram que a TFC foi eficaz na diminuição da espessura dos tecidos moles.		
Devoogdt et al.^19^ (Bélgica)	Participaram do presente estudo 42 pacientes com dissecção axilar unilateral por câncer de mama primário, sendo avaliadas para a evolução de quadros de linfedema secundário a câncer de mama.	1b	O ultrassom foi realizado a fim de investigar a evolução da espessura e ecogenicidade da cútis e subcutânea até um ano após dissecção axilar por câncer de mama e comparar pacientes com e sem linfedema objetivo. As avaliações ultrassonográficas de ambos os braços foram realizadas imediatamente e 6 e 12 meses após a cirurgia axilar. Em cada local de medição, foi feita uma imagem sagital e transversal. O ponto de referência marcado estava localizado no meio da sonda, no qual uma quantidade mínima de pressão foi aplicada.	Na avaliação ultrassonográfica, a ecogenicidade do subcutâneo foi mais frequentemente perturbada no lado afetado (em 7-33% dos pacientes) do que no lado saudável (0-19%). A prevalência de ecogenicidade alterada do subcutâneo no braço afetado foi (não significativa) clinicamente relevante e diferente entre pacientes com e sem linfedema no punho, antebraço dorsal e bíceps e tríceps. Pelo presente estudo, parece que o aumento da espessura do subcutâneo do antebraço ventral e do tríceps e a alteração da ecogenicidade da cútis no punho são bons indicadores para identificar um paciente com linfedema	Eficaz: em pacientes com câncer de mama, a ultrassonografia pode ser útil para diagnosticar linfedema de braço; no entanto, não pode ser usada como um teste de diagnóstico separado.	Ultrassom (Siemens Acuson Antares Premium, Erlangen, Alemanha) com sonda linear de alta frequência de 13 MHz.
Abreu et al.^20^ (Brasil)	80 pacientes submetidas à mastectomia e radioterapia e alocadas em dois grupos: 40 pacientes com LSCM e 40 sem a doença.	1b	Foram pesquisadas alterações ultrassonográficas nos sentidos transverso e longitudinal.	Prevalência global de 83,8% de alterações ultrassonográficas na veia axilar das portadoras de LSCM.	Eficaz: a prevalência de alterações ultrassonográficas foi maior nas pacientes com LSCM.	Equipamento de ultrassonografia modelo Sonoace X8 ou SA 8000EX Prime, com transdutor linear multifrequencial de 5 a 12 MHz, ambos da Medison Co. Ltd., 1003 Daechi-dong, Gangnam-gu, Seoul 135-280 Coreia do Sul.
Bok et al.^5^ (Coreia do Sul)	32 pacientes com linfedema relacionado ao câncer de mama foram randomizados: um grupo recebeu TFC + PRE e o outro apenas PRE.	1b	A espessura do tecido subcutâneo e músculo foi medida pela USD.	A USD é uma boa ferramenta para medir a mudança na espessura do músculo após PRE para confirmar o efeito do tratamento do linfedema. Pode ser usada como diagnóstico para linfedema e também como um método para determinar o tratamento.	Eficaz: foi possível avaliar pela USD os efeitos do PRE no tratamento de pacientes com LSCM.	Ultrassom (MyLab 50, Esaote, Itália).
						Não informada a frequência do equipamento.
			Medição em dois pontos: 1) parte proximal, 10 cm proximal ao ponto do cotovelo; e 2) parte distal, 10 cm distal ao ponto do cotovelo.			
Johnson et al.^21^ (Estados Unidos)	17 mulheres com linfedema secundário ao câncer de mama.	1b	O ultrassom foi colocado em dois locais em todas as extremidades superiores envolvidas e não envolvidas pelo linfedema.	A imagem do USD é confiável para medir a entropia média entre membros envolvidos e não envolvidos no antebraço anterior. Comparando com avaliação clínica do edema, demonstrou boa correlação para entropia na parte inferior do braço posterior.	Eficaz: a imagem da USD como uma ferramenta para quantificar tecidos subcutâneos é um método seguro, móvel e eficaz para medir a textura do tecido do linfedema.	Aparelho de ultrassom Sonosite M-Turbo com transdutor linear de 15 MHz.
			Foram colhidas 55 medidas para cada local.			
Suehiro et al.^22^ (Japão)	Participaram do estudo 30 pacientes com linfedema em braço relacionado ao câncer de mama em estágio II unilateral.	1b	A ultrassonografia foi realizada para investigar a espessura da pele, SELEB e SCT, bem como o grau de aumento na SEG e SEFS em braços com linfedema. A pele e o tecido subcutâneo de ambos os braços de 30 pacientes com linfedema relacionado ao câncer de mama em estágio II unilateral foram examinados em cinco pontos (braço medial/lateral superior/antebraço e dorso da mão). Os graus de SEG e SEFS foram determinados de acordo com a gravidade (intervalo: 0-2).	Todos os parâmetros medidos, exceto o SEFS no braço medial, foram significativamente maiores no lado com linfedema do que no lado normal. Os parâmetros diferiram mais notavelmente no antebraço medial. Não foi possível confirmar um aumento nas notas SEG/SEFS de acordo com a gravidade, ou seja, notas SEG/SEFS mais altas no antebraço do que no braço.	Eficaz: o ultrassom apresentou boa capacidade de demonstrar a espessura da pele, SELEB e SCT, bem como os graus SEG e SEFS em braços com linfedema e braços normais de pacientes com LSCM, evidenciando ainda que o aumento desses parâmetros foi maior no antebraço medial do braço com linfedema.	Sistema de ultrassom (LOGIQ S6; GE Healthcare, Little Chalfont, Buckinghamshire, Reino Unido) com um transdutor linear de 7 a 12 MHz.
Dai et al.^23^ (Japão)	10 MMSS com LSCM com história de ADLA e 14 MMSS com LSCM.	1c	Calculada a assimetria a partir da análise de histograma na ROI definida nas imagens da derme, usando a mesma técnica para ambos os membros.	A distribuição de colágeno na camada papilar da derme foi diferente após os episódios de ADLA, com base nos resultados de assimetria e alta ecogenicidade de pixels.	Eficaz: a USD é eficaz na identificação das alterações estruturais da ADLA, fator de risco para aumento do linfedema.	Ultrassom Derma scan C (Cortex Technology, Smedevaenget, Dinamarca) a 20 MHz.
				A USD é útil para avaliar a assimetria e confirmação da estrutura dérmica.		
Hansdorfer-Korzon et al.^24^ (Polônia)	Foram incluídas na pesquisa 35 mulheres com LSCM, e 29 terminaram o estudo.	1b	A ultrassonografia (modo B) foi usada para avaliar o linfedema na lateral do tórax após a mastectomia. Esse teste foi realizado três vezes em um local específico do lado operado e simetricamente no lado oposto. Posteriormente, os pacientes foram ajustados com um espartilho de compressão apropriado e reavaliados novamente com a ultrassonografia.	A ultrassonografia identificou as alterações subcutâneas provenientes do linfedema.	Eficaz: o ultrassom foi eficaz na avaliação dos efeitos da terapia proposta.	Ultrassom (Voluson E8, a sonda ML6-15; GE Healthcare, Piscataway NJ, EUA). Não foram informados o transdutor e a frequência usados.
Jeon et al.^3^ (Coreia do Sul)	32 pacientes com LSCM randomizados em 2 grupos: PRE e não PRE.	1b	As espessuras do músculo e tecido subcutâneo foram mensuradas pela USD. As espessuras do tecido muscular e subcutâneo foram medidas na linha de base, 4 semanas e 8 semanas após o PRE.	A espessura inicial do músculo de todos os participantes foi significativamente menor no braço linfedematoso em comparação ao MMSS não afetado. O tecido subcutâneo no MMSS com LSCM era mais espesso.	Eficaz: a USD é uma das melhores ferramentas para diagnosticar e determinar a eficácia do tratamento no LSCM.	Não informado.
Yang et al.^13^ (Estados Unidos)	A viabilidade clínica foi testada com quatro participantes: dois pacientes com LSCM e dois voluntários saudáveis.	1c	Método de imagem de deformação 2D, usando o registro de imagens de modo B de ultrassom pré- e pós-compressão. O teste foi através de uma série de experiências usando elastografia sob várias pressões.	Os resultados iniciais são encorajadores, e é necessário um grande estudo clínico para avaliar ainda mais essa tecnologia de imagem por ultrassom 2D.	Eficaz: a USD 2D foi eficiente na identificação das alterações causadas por linfedema de MMSS.	Scanner clínico (SonixTouch, Ultrasonix, British Columbia, Canadá) com um transdutor de matriz linear (L14-5 / 38). Frequência central de 10 MHz.
Hashemi et al.^25^ (Canada)	Foram avaliadas 7 mulheres com LSCM estágio 2.	1b	A USD identificou as propriedades dos tecidos em mulheres com LSCM.	A avaliação ultrassonográfica na elastografia foi eficaz e estadia o LSCM, além de avaliar o tecido.	Eficaz: com a aplicação de novas técnicas na elastografia pela USD, é possível avaliar melhor o LSCM e fornecer tratamentos direcionados para reduzir a progressão dessa condição.	Ultrassom sistema Alpinion E-Cube (Bothell, WA, EUA) usando o transdutor L3-8 na frequência central de 10 MHz e taxa de amostragem de 40 MHz.
			Usadas técnicas de elastografia por USD quase estática para investigar sua utilidade no estadiamento do linfedema.			
Yang et al.^26^ (Coreia do Sul)	158 mulheres com no mínimo 6 meses após o tratamento para câncer de mama unilateral e com ou sem linfedema foram recrutadas, retrospectivamente.	1b	Com o uso da USD, a ecogenicidade subcutânea foi avaliada no lado medial do braço e antebraço de ambos os lados e graduada por grau de ecogenicidade no subcutâneo.	O uso da USD é indicado na avaliação do linfedema, principalmente na região medial do antebraço.	Eficaz: a ecogenicidade subcutânea do ultrassom pode melhorar a precisão do diagnóstico de linfedema no antebraço.	Ultrassom equipado com um transdutor de 11 MHz.
						Não foi informado qual o equipamento utilizado.
Iyigun et al.^27^ (Turquia)	36 pacientes do sexo feminino, com linfedema estágio 1 ou 2 em membros superiores secundário a câncer de mama.	1b	A ultrassonografia foi realizada com a finalidade de se fazer um total de três medições do braço com linfedema e extremidade normal, sendo uma a 10 cm proximal da apófise estiloide da ulna para o antebraço e outra a 10 cm proximal do epicôndilo medial para o braço. As imagens foram obtidas de 10 regiões subcutâneas diferentes, usadas para calcular as velocidades médias das ondas de cisalhamento.	O uso do ultrassom na técnica de elastografia por onda de cisalhamento foi capaz de identificar áreas com linfedema.	Eficaz: ultrassonografia é uma ferramenta útil na distinção e diagnóstico dos estágios iniciais e tardios do linfedema.	Ultrassom SWE (Acuson S 3000 US) transdutor de 9L4, com frequência no intervalo de 4-9 MHz.
Mander et al.^28^ (Estados Unidos/Itália)	287 mulheres com LSCM.	1a	A espessura tecidual foi medida e comparada considerando o membro contralateral como controle. O membro foi considerado afetado pelo linfedema se tivesse duas medidas consecutivas de circunferências maiores que 2 cm em comparação com membro contralateral.	A USD tradicional pode fornecer uma caracterização secundária do LSCM, com mapeamento relacionado e dados úteis para uma melhor compreensão da fisiopatologia linfática e para um adequado protocolo terapêutico.	Eficaz: a USD se mostrou eficaz em realizar a caracterização do LSCM.	Ultrassom (Sono Scape S22, sonda linear 12L-A, 192 elementos, 6-16 MHz).
Polat et al.^29^ (Turquia)	41 mulheres com história de cirurgia de mama unilateral e dissecção axilar ou excisão de linfonodo sentinela.	1b	A espessura e rigidez do tecido cutâneo e subcutâneo do antebraço e braço foram medidos por ultrassom e SWE. O membro afetado e dos membros contralaterais foram comparados.	No grupo linfedema latente, as medidas de espessura do tecido cutâneo do antebraço afetado e do tecido cutâneo e subcutâneo do braço afetado foram significativas.	Eficaz: a USD foi eficaz para diagnosticar o LSCM mesmo em estágio latente.	Ultrassom Modo B - sistema Acuson S2000 US (Siemens Medical Solutions, Mountain View, CA, EUA) equipado com uma placa de 9 MHz
Suehiro et al.^30^ (Japão)	120 pacientes que se submeteram à cirurgia de câncer de mama e foram acompanhadas quanto ao aparecimento de linfedema.	1b	A ultrassonografia de pele e tecido subcutâneo foi utilizada avaliando a ecogenicidade dos membros avaliados com o objetivo de evidenciar sua capacidade diagnóstica para a detecção precoce do linfedema pós-mastectomia de 1 mês no pré-operatório até 2 anos no pós-operatório. Avaliado o aumento difuso da ecogenicidade na camada subcutânea; e linhas ecogênicas.	O ultrassom apresentou evidências de alterações da ecogenicidade subcutânea entre as regiões avaliadas nos membros superiores avaliados para o desenvolvimento do linfedema secundário ao câncer de mama.	Eficaz: a ultrassonografia foi capaz de identificar áreas com aumento da densidade celular e aumento de conteúdo de colágeno no tecido, o que indica a presença de inflamação subcutânea, situação que evidencia a presença de linfedema.	Ultrassom Sistema Logiq S6 (GE Healthcare, Little Chalfont, Buckinghamshire, Reino Unido) com um transdutor linear de 7 a 12 MHz.
Giray e Yağcı^8^ (Turquia)	50 mulheres com linfedema de braço relacionado ao câncer de mama.	1b	O ultrassom foi utilizado para avaliar a confiabilidade inter e intraexaminadores no diagnóstico de linfedema através da identificação do grau de ecogenicidade subcutânea e o grau de espaço livre de eco subcutâneo, que permitem a semiquantificação da inflamação inespecífica do tecido subcutâneo e o acúmulo de líquido no linfedema relacionado ao câncer de mama. A sonda foi mantida em posição axial no antebraço medial sobre o músculo flexor radial do carpo. A profundidade da imagem capturada foi fixada em 2 cm.	A ultrassonografia evidenciou que grau de SEG e o grau de SEFS são ambos confiáveis na avaliação intra e interexaminadores, no entanto, deve-se ter em mente que os avaliadores concordam menos ao classificar SEG de pacientes com estágio clínico intermediário, e os avaliadores concordam mais ao classificar o grau de SEFS de pacientes com estágio clínico intermediário.	Eficaz: com base nas descobertas desse estudo, SEG e SEFS demonstraram confiabilidade aceitável. O sistema de classificação SEG e SEFS com ultrassom pode ser útil para acompanhar a progressão, a composição e o manejo do linfedema relacionado ao câncer de mama.	Aparelho de ultrassom Esaote MyLab com sonda de matriz linear de 6-18 MHz.
Hashemi et al.^31^ (Canadá)	A população do estudo foi composta por 7 mulheres com linfedema de estágio 2 relacionado ao câncer de mama.	1c	A ultrassonografia pelo método da elastografia foi usada para comparar as propriedades mecânicas dos braços afetados e não afetados para fornecer uma alternativa à avaliação subjetiva atual do linfedema relacionado ao câncer de mama. Esse método foi comparado ao teste de corrosão habitualmente usado para a finalidade de avaliação de linfedema. Foram coletados dados de ultrassom de ambos os braços de sete pacientes com estágio 2 linfedema, em seis locais diferentes em cada braço a fim de identificar mudanças nas propriedades mecânicas do tecido relacionadas à detecção e estadiamento de linfedema.	A utilização da elastografia por ultrassom permitiu obter uma visão sobre as diferenças das propriedades no tecido entre o membro não afetado (não linfedematoso) em comparação com o membro afetado (membro linfedematoso). Os valores de taxas de deformação no membro afetado são consistente e significativamente mais baixos na pele que em gordura subcutânea e camadas de músculo esquelético. Taxas de deformação foram encontradas mais baixas na pele no afetado em comparação com o membro não afetado.	Eficaz: a técnica de elastografia proposta é mais sensível do que o teste de corrosão.	Ultrassom sistema Alpinion E-Cube (Bothell, WA, EUA) usando o transdutor L3-12H na frequência central de 10 MHz e a taxa de amostragem de 40 MHz.
Seo et al.^7^ (Coreia do Sul)	6 mulheres que foram submetidas à cirurgia de câncer de mama e foram diagnosticadas com linfedema de membro superior unilateral.	1c	A ultrassonografia foi utilizada para avaliar os efeitos da intervenção com DLM, sendo realizada nos períodos de pré-intervenção e pós-intervenção, sendo realizada avaliação volumétrica tanto do lado afetado quanto do lado contralateral, comparando-os.	A imagem de ultrassom apresentou diferenças significativas no volume do membro afetado comparado ao lado não afetado. No lado afetado, embora a ultrassonografia tenha mostrado uma diminuição significativa após a DLM, não houve diferença significativa quando comparada ao basal.	Eficaz: a ultrassonografia mostrou-se eficaz na avaliação na abordagem terapêutica realizada (DLM).	Ultrassom (MySono U5; Samsung Medison Co., Seul, Coreia do Sul) com transdutor linear de 7,5 MHz.
Niwa et al.^32^ (Japão)	Esse estudo incluiu 20 mulheres que foram tratadas para câncer de mama unilateral e que, posteriormente, desenvolveram linfedema em membro superior.	1b	O tecido subcutâneo foi escaneado por meio de um sistema ultrassom usando um transdutor linear de 6 a 15 MHz para avaliar a capacidade das características de textura para discriminar a presença de fluido acumulado no tecido subcutâneo do linfedema secundário ao câncer de mama. O acúmulo de fluido foi observado usando um sistema MR de 3 Tesla em condições de estado estacionário de eco duplo.	Houve uma diferença significativa entre os três grupos (com área hiperintensa, sem área hiperintensa e lado não afetado) em características texturais, revelando diferenças significativas em sete características texturais dentro da área hiperintensa, sendo possível discriminar a presença de fluido acumulado no tecido subcutâneo de LSCM usando imagens de ultrassom.	Eficaz: esse estudo revelou que sete características de textura quantificadas por dados de imagem de US podem fornecer informações sobre o acúmulo de líquido no tecido subcutâneo do linfedema.	Ultrassom Sonosite Edge II; Sonosite, Inc., FUJIFILM) usando um transdutor linear de 6 a 15 MHz.
Kim et al.^33^ (Coreia do Sul)	69 pacientes do sexo feminino com diagnóstico de estágio 1 de linfedema relacionado a câncer de mama avançado.	1b	A ultrassonografia foi realizada em ambos os braços de cada sujeito, com os pacientes deitados. O examinador marcou as áreas para medir a seção transversal desejada.	O método de medida da seção transversa apresentou coeficientes elevados na avaliação do linfedema. A dureza dos tecidos moles, que reflete o estado histológico, pode ser medida e mostra características diferentes dos tecidos com o mesmo volume de linfedema.	Eficaz: a combinação desses dois métodos ultrassonográficos parece refletir não apenas alterações estruturais, mas também alterações histológicas nos tecidos moles após a ocorrência do linfedema.	Ultrassom (LOGIQ E9; General Electric, Boston, MA, EUA) com transdutor de 7,5 MHz.
			A rigidez do tecido subcutâneo também foi obtida medindo as diferenças de espessura do tecido mole ao aplicar pressão mínima e máxima na pele (complacência) e sua relação com a espessura inicial.			
Erdinç Gündüz et al.^34^ (Turquia)	34 pacientes do sexo feminino com linfedema secundário a câncer de mama. Linfedema unilateral relacionado ao câncer de mama.	1b	Realizada avaliação ultrassonográfica avaliando as espessuras cutâneas e subcutâneas, sendo medidas em quatro quadrantes nos pontos marcados, e também as alterações do tecido subcutâneo foram graduadas de acordo com a escala de grau de ecogenicidade subcutânea ultrassonograficamente. Realizada medição ultrassonográfica: ecogenicidade ultrassonográfica subcutânea e medição da espessura da pele e subcutânea.	A gravidade do linfedema foi graduada ultrassonograficamente de acordo com a escala SEG em estágio 0, estágio 1 e estágio 2 avaliando as linhas ecogênicas de ecogenicidade A avaliação ultrassonográfica apresentou diferença entre as duas extremidades superiores, apresentou uma sensibilidade alta (0,83%) e uma especificidade aceitável (0,75%) na diferenciação de linfedema de Grau II e Grau III.	Eficaz: foi estabelecida uma correlação entre as medidas circunferenciais e as medidas ultrassonográficas. A ultrassonografia pode ser usada complementarmente às medidas circunferenciais no diagnóstico de linfedema.	Não informado.
Duyur Çakit et al.^16^ (Turquia)	Foram incluídos no estudo 47 pacientes com linfedema secundário a câncer de mama em membro superior unilateral.	1b	O ultrassom foi realizado a fim de determinar o seu papel no acompanhamento da eficácia da CDT em diferentes subgrupos de pacientes com linfedema relacionado ao câncer de mama. Todos os pacientes foram submetidos a CDT, as medidas de circunferência e avaliações ultrassonográficas da espessura dos tecidos moles foram realizadas em dois locais anatômicos, e os volumes dos membros superiores foram calculados usando a fórmula do cone truncado antes e depois da CDT.	Houve diminuições significativas em ambas as medidas circunferenciais e espessuras ultrassonográficas dos tecidos moles em pacientes não obesos e pacientes com linfedema estágio 2 após 15 sessões de CDT. Os valores ultrassonográficos da espessura do tecido mole foram correlacionados com os valores da circunferência do braço e antebraço antes e depois da CDT.	Eficácia relativa: a ultrassonografia é um método confiável para medir a espessura do tecido mole e a eficácia do tratamento após a CDT apenas em pacientes não obesos e em estágio 2 com LSCM.	Sistema de Ultrassom com transdutor linear de 7-12 MHz, Logic P5, GE medical systems, Wisconsin, EUA.

MMSS = membros superiores; USD = ultrassonografia diagnóstica; LSCM = linfedema relacionado ao câncer de mama; TFC = terapia física complexa; PRE = exercício resistido progressivo; ADLA = dermatolinfangioadenite aguda; DLM = drenagem linfática manual; SELEB = banda subepidérmica baixa ecogênica; SCT = tecido subcutâneo; SEG = ecogenicidade subcutânea; SEFS = espaço livre de eco subcutâneo; ROI = região de interesse; CDT = terapia descongestiva complexa; SWE = elastografia de onda de cisalhamento.

Polat et al.^29^ e Iyigun et al.^27^ relataram sobre o tempo possível de diagnóstico: considerando a realização da USD factível em estado latente, estágios iniciais e tardios. Além disso, Yang et al.^26^ correlacionaram a ecogenicidade da onda ultrassonográfica como forma de melhorar o diagnóstico do linfedema.

Duyur Çakit et al.^16^ discorreram sobre uso da USD no acompanhamento da eficácia da terapia descongestiva complexa em diferentes subgrupos e sugeriram a eficácia relativa do instrumento, apontando que pacientes não obesos e em estágio 2 com LSCM podem ser avaliados com maior veracidade.

O processo de formação do linfedema envolve o aumento da atividade de neutrófilos, macrófagos e fibroblastos e a inflamação e deposição de colágeno (fibrose). Conforme Suehiro et al.^30^ , através da USD é possível captar o aumento do colágeno e o aumento da inflamação subcutânea, enquanto segundo Kim et al.^33^ , também é possível notar alterações histológicas além das estruturais. Por fim, Han et al.^18^ descrevem a fibrose como um achado ultrassonográfico de grande valor, além da extensão do edema.

Entre os 25 artigos selecionados de 2004 a 2022, sete apresentaram considerações sobre o uso da USD em conjunto com técnicas terapêuticas com o intuito de demonstrar o progresso ou regresso obtido no tratamento. Seo et al.^7^ utilizaram a drenagem linfática manual (DLM) como método para tratamento do linfedema e relataram a eficácia da USD no processo. Já Devoogdt et al.^19^ sugerem a necessidade de outro método diagnóstico em conjunto até que haja maior evidência científica.

A elastografia realizada durante a USD é um método capaz de identificar a rigidez no tecido, observando a presença de possíveis nódulos e sendo indolor e pouco invasivo. Quatro autores apontaram a eficácia da elastografia. Hashemi et al.^25^ apontaram a importância do método não só no diagnóstico, mas também no estadiamento de LSCM. Hashemi et al.^31^ descreveram a elastografia como mais sensível que o teste de corrosão.

Os equipamentos utilizados variaram entre diferentes modelos e frequências. Mellor et al.^17^ e Dai et al.^23^ utilizaram o equipamento Dermascan (20 MHz). Quatro artigos não especificaram o equipamento utilizado, e Bok et al.^5^ e Hansdorfer-Korzon et al.^24^ não informaram a frequência usada. Han et al.^18^ e Suehiro et al.^22^ utilizaram o modelo Logig. Os demais instrumentos e frequências não coincidiram.

Os pacientes e os estudos variaram de diversas formas e não mantiveram um padrão. Foram encontrados 11 artigos de estudo transversal, sendo dois transversais de acurácia e um descritivo transversal, enquanto foram identificados 11 artigos longitudinais prospectivos e um apenas longitudinal retrospectivo. Han et al.^18^ e Abreu et al.^20^ separaram os participantes em dois grupos, o primeiro de pacientes com LSCM e o segundo de pacientes saudáveis, no entanto, os dois variaram na quantidade de pacientes em cada grupo.

A Figura 2 demonstra de forma esquemática a diferença de um tecido normal e de um tecido com LSCM avaliado pela USD.

**Figura 2:** Representação do tecido normal e do tecido linfedematoso.

A Tabela 1 também apresenta o grau de evidência de cada artigo. Nesta revisão sistemática, foram incluídos artigos experimentais, com níveis de evidência em sua maioria 1b e 1c, de acordo com a Escala de Oxford de graus de evidência. A maioria das evidências disponíveis está no nível 1, sendo o procedimento recomendado (grau de recomendação A).

## DISCUSSÃO

Esse estudo realizou uma ampla revisão a partir de 25 artigos científicos que registram a utilização da USD como método diagnóstico em casos de linfedema. Há um consenso sobre a eficácia do instrumento para identificar a presença do edema no tecido subcutâneo.

Atualmente, não existe uma ferramenta específica para o diagnóstico em estágios iniciais, quando os sintomas ainda não apareceram. Os testes mais populares para caracterizar o LSCM incluem os seguintes: medição da circunferência do braço; perometria, que avalia o volume do braço afetado em comparação com o braço não afetado; e bioimpedância, que verifica a resistência a correntes elétricas indolores passadas pelo braço^35^ . Outros métodos de imagem usados para avaliar a LSCM são a tomografia computadorizada, a ressonância magnética e a linfografia com verde de indocianina; no entanto, eles não são portáteis e são mais caros do que os outros^8^ . Por outro lado, a técnica de imagem de linfocintilografia é considerada o padrão de critério para o diagnóstico de LSCM, usando uma substância radiomarcada para visualizar o sistema linfático e revelar a presença e o calibre dos vasos linfáticos, linfonodos, colaterais e atraso na captação de radionuclídeos. No entanto, esse método geralmente não é preferido devido à falta de um protocolo padrão, à invasão do procedimento e à exposição do paciente à radiação^35^ .

A ultrassonografia é um procedimento seguro, fácil e de baixo custo para avaliar pacientes com LSCM. As alterações incluem o aumento da espessura da derme, a mudança de hipoecogenicidade para hiperecogenicidade do subcutâneo e a retenção de líquidos na derme, no espaço interlobular e na fáscia superficial. Embora essas alterações possam ser difíceis de detectar em imagens de ultrassom, elas podem fornecer uma medida quantitativa da espessura do tecido cutâneo, fascial e circundante para avaliar a LSCM^35^ .

A USD também pode ser útil como uma ferramenta prognóstica eficaz, pois pode permitir a identificação de pacientes com risco de desenvolver uma resposta patológica incompleta. A aplicação dessa técnica de imagem poderia reduzir o tempo gasto na realização de vários procedimentos diagnósticos invasivos e também poderia reduzir os custos de saúde envolvidos no processo^36^ . Os parâmetros morfológicos e funcionais encontrados na USD podem ser correlacionados com o diagnóstico, o estágio, o prognóstico e o efeito terapêutico clínicos do LSCM^37^ .

A ultrassonografia é considerada um exame de imagem fácil e seguro para avaliar a espessura da pele e do tecido subcutâneo e, como resultado, tem sido estudada para avaliar pacientes com linfedema. Nos últimos anos, a elastografia por ultrassom tem sido usada para avaliar o LSCM; no entanto, os parâmetros para avaliar, diagnosticar e estadiar a doença ainda não foram bem definidos.

A USD parece ser um método vantajoso por ser seguro, pouco invasivo, prático, de baixo custo, sem uso de radiação ionizante, podendo ser utilizado em pré-, intra e pós-operatórios, além de ser útil para avaliar a eficácia de terapias no tratamento do linfedema. As desvantagens observadas foram a necessidade de um operador com habilidade para o procedimento, a aplicação da pressão correta durante o uso da USD e a necessidade de mais estudos sobre o assunto.

Por fim, a USD na prática clínica parece ser um recurso promissor para a mensuração, classificação e evolução do LSCM de forma objetiva. O procedimento é rápido, indolor, prático e pouco invasivo para os pacientes, além de ser possível encontrar o instrumento em vários locais de atendimento médico.

## CONCLUSÃO

Em virtude dos fatos mencionados, entende-se que a ultrassonografia como instrumento de avaliação em casos de linfedema secundário ao câncer de mama apresentou-se necessária, visto que se mostrou eficaz de forma mais objetiva, sendo um recurso viável para diagnósticos.
